# Publisher Correction: Light absorption enhancement of black carbon in a pyrocumulonimbus cloud

**DOI:** 10.1038/s41467-024-52857-7

**Published:** 2024-10-10

**Authors:** Payton Beeler, Joshin Kumar, Joshua P. Schwarz, Kouji Adachi, Laura Fierce, Anne E. Perring, J. M. Katich, Rajan K. Chakrabarty

**Affiliations:** 1 Center for Aerosol Science and Engineering, Department of Energy, Environmental and Chemical Engineering, Washington University in St. Louis, St. Louis, MO USA; 2 Atmospheric, Climate, and Earth Sciences Division, Pacific Northwest National Laboratory, Richland, WA USA; 3 National Oceanic and Atmospheric Administration (NOAA) Chemical Sciences Laboratory (CSL), Boulder, CO USA; 4 Department of Atmosphere, Ocean and Earth System Modelling Research, Meteorological Research Institute, Tsukuba, Japan; 5 Department of Chemistry, Colgate University, Hamilton, NY USA; 6 Cooperative Institute for Research in Environmental Sciences, University of Colorado, Boulder, CO USA; 7 Present Address: BAE Systems, Inc, Boulder, CO USA

**Keywords:** Climate and Earth system modelling, Atmospheric dynamics

Correction to: *Nature Communications* 10.1038/s41467-024-50070-0, published online 25 July 2024

The copyright holder for this article was incorrectly given as ‘The Author(s)’ but should have been ‘Battelle Memorial Institute, The Authors, MRI, Ball Aerospace. Parts of this work were authored by US Federal Government authors and are not under copyright protection in the US; foreign copyright protection may apply’. This has been corrected in both the PDF and HTML versions of the Article.

