# Supplementary material for: Light absorption enhancement of black carbon in a pyrocumulonimbus cloud
Source: Nat Commun. 2024 Jul 25;15:6243. doi: 10.1038/s41467-024-50070-0 (PMC11272781; doi:10.1038/s41467-024-50070-0)
Supplement: Supplementary file 1 — Supplementary Information [file 41467_2024_50070_MOESM1_ESM.pdf]

Supplementary Information for:

Light Absorption Enhancement by Black Carbon in a Pyrocumulonimbus Cloud

Payton Beeler, Joshin Kumar, Joshua P. Schwarz, Kouji Adachi, Laura Fierce, Anne E. Perring, J.M. Katich, and Rajan K. Chakrabarty

Supplementary Figures:

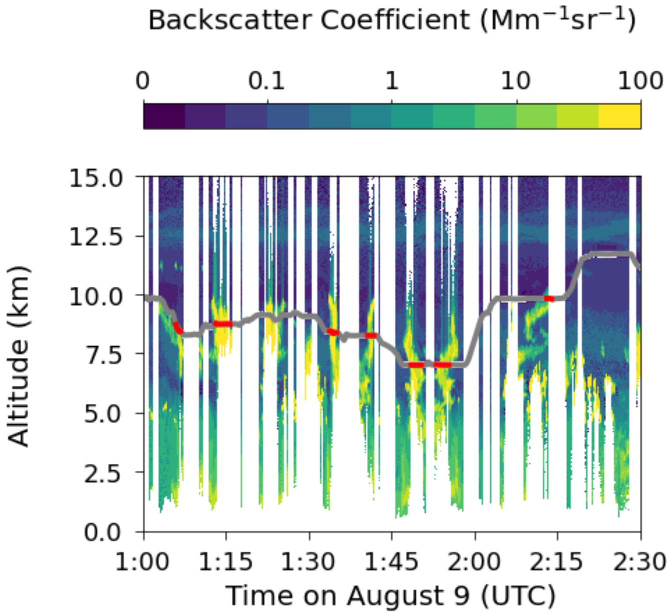

Figure S1. Time series of backscatter coefficient measured by the up-and-down viewing DIAL-HSRL on board the DC-8 aircraft. The location of the aircraft is shown in grey, and intersections of the pyroCb cloud are indicated by red portions of the flight path. Large gaps correspond to steep turns, during which the DIAL-HSRL was turned off. Areas of high backscattering indicate the vertical location of the pyroCb cloud, showing that the pyroCb cloud is located at altitudes between 7 and 10 km.

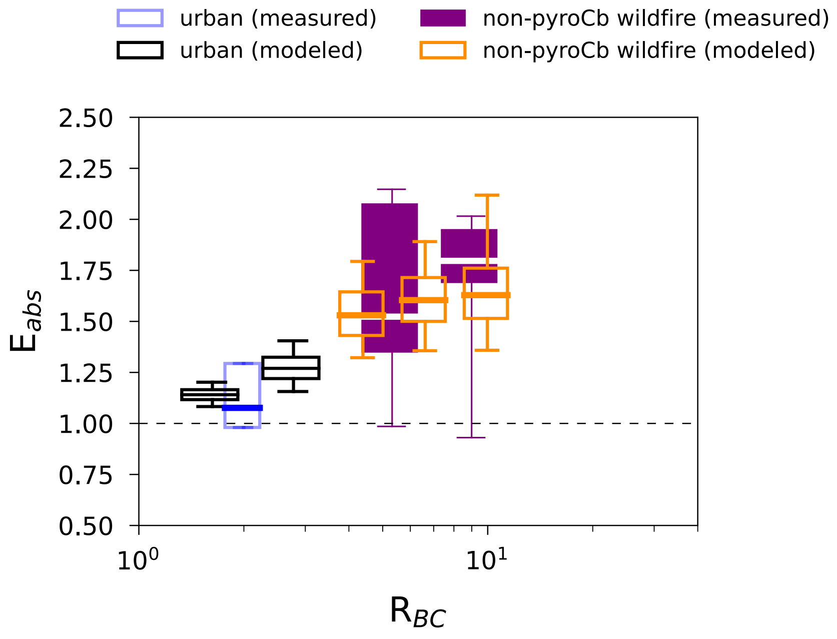

Figure S2. Comparison of measured and modeled absorption enhancement for urban and wildfire BC. We find good agreement between measured and modeled values, and therefore assume that modeled absorption enhancement is an accurate representation of actual light absorption enhancement by BC from all sources.

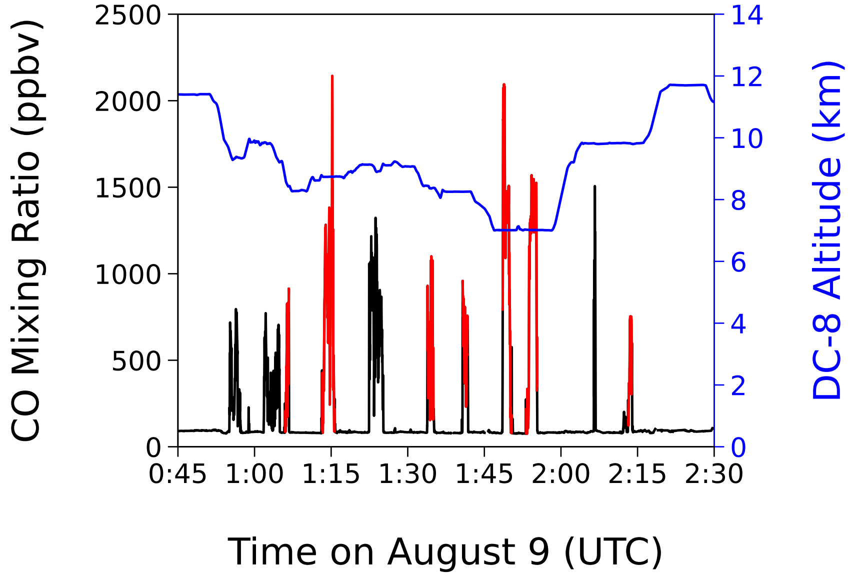

Figure S3. Time series of CO concentration measured during pyroCb cloud intersects (black). Altitude of DC-8 aircraft is shown in blue. This study uses data from portions of the DC-8 flight with CO concentration > 500 ppb and > 100 BC particles detected by the SP2. PyroCb cloud transects used for analysis are indicated by red highlights.

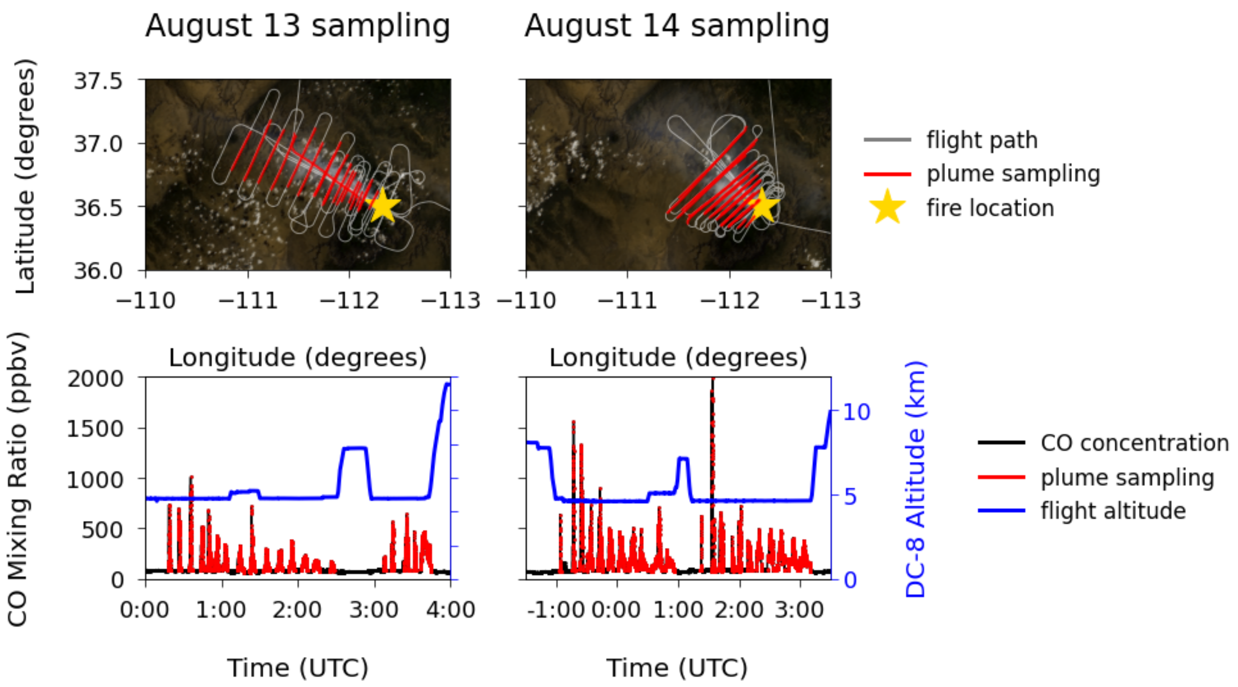

Figure S4. Overview of FIREX-AQ sampling of the Castle/Ikes wildfires on August 13 (left column) and August 14 (right column). Top row shows GOES-16 satellite image with DC8 flight path (grey). Portions of the flight designated as plume sampling are highlighted in red. Bottom row shows time series of measured CO concentration (right axis) and DC8 aircraft altitude (right axis). Elevated CO concentration is used to distinguish plume sampling times, and are highlighted in red.

Supplementary Tables

Table S1. Median SP2-measured per-particle R_{BC} and per-particle BC mass in wildfire plumes. The microphysical BC properties in the Castle/Ikes wildfire are not significantly different than those of other wildfires. We therefore only include data from the Castle/Ikes wildfire in the main text, due to detailed knowledge of non-BC absorption from these fires.

Fire Name	Median Per-Particle R_{BC}	Median Per-Particle BC Mass (fg)	Particles Detected
Sheep	7.41	1	252
Shady	13.01	1.08	5362
Northhills	8.79	1.27	3332
Tucker	9.66	0.96	7750
Tucker/Milepost97	11.91	1.2	2456
Lefthand	7.48	1.31	7809
Ridge Top	5.58	1.78	4302
Lick Creek	9.58	1.24	2828
Castle	7.74	1.55	21434
Ikes	8.4	1.12	34
AZ Complex	5.61	1.67	83
Sheridan	8.45	1.08	48819
Saber	8.18	1.24	193
Boulin	4.72	1.86	215
Sheridan/Boulin	6	1.57	84
